# Arginase 1 Insufficiency Precipitates Amyloid-*β* Deposition and Hastens Behavioral Impairment in a Mouse Model of Amyloidosis

**DOI:** 10.3389/fimmu.2020.582998

**Published:** 2021-01-14

**Authors:** Chao Ma, Jerry B. Hunt, Maj-Linda B. Selenica, Awa Sanneh, Leslie A. Sandusky-Beltran, Mallory Watler, Rana Daas, Andrii Kovalenko, Huimin Liang, Devon Placides, Chuanhai Cao, Xiaoyang Lin, Michael B. Orr, Bei Zhang, John C. Gensel, David J. Feola, Marcia N. Gordon, Dave Morgan, Paula C. Bickford, Daniel C. Lee

**Affiliations:** ^1^Department of Molecular Pharmacology and Physiology, Morsani College of Medicine, University of South Florida, Tampa, FL, United States; ^2^Sanders-Brown Center on Aging, Department of Neuroscience, College of Medicine, University of Kentucky, Lexington, KY, United States; ^3^Department of Pharmaceutical Sciences, College of Pharmacy, University of South Florida, Tampa, FL, United States; ^4^Sanders-Brown Center on Aging, Department of Molecular and Cellular Biochemistry, College of Medicine, University of Kentucky, Lexington, KY, United States; ^5^Spinal Cord and Brain Injury Research Center, Department of Physiology, College of Medicine, University of Kentucky, Lexington, KY, United States; ^6^Center for Neurogenetics, Feil Family Brain and Mind Research Institute, Weill Cornell Medicine, Cornell University, New York, NY, United States; ^7^Department of Pharmacy Practice and Science, College of Pharmacy, University of Kentucky, Lexington, KY, United States; ^8^Department of Translational Neuroscience, College of Human Medicine, Michigan State University, Grand Rapids, MI, United States; ^9^Center of Excellence for Aging and Brain Repair, Department of Neurosurgery and Brain Repair, Morsani College of Medicine, University of South Florida, Tampa, FL, United States; ^10^Research Service, James A. Haley Veterans Affairs Hospital, Tampa, FL, United States

**Keywords:** Alzheimer’s disease, neuroinflammation, arginine metabolism, macrophage, microglia, phagocytosis, cognition, Tg2576

## Abstract

Alzheimer’s disease (AD) includes several hallmarks comprised of amyloid-*β* (Aβ) deposition, tau neuropathology, inflammation, and memory impairment. Brain metabolism becomes uncoupled due to aging and other AD risk factors, which ultimately lead to impaired protein clearance and aggregation. Increasing evidence indicates a role of arginine metabolism in AD, where arginases are key enzymes in neurons and glia capable of depleting arginine and producing ornithine and polyamines. However, currently, it remains unknown if the reduction of arginase 1 (*Arg1)* in myeloid cell impacts amyloidosis. Herein, we produced haploinsufficiency of *Arg1* by the hemizygous deletion in myeloid cells using *Arg1^fl/fl^* and *LysMcre^Tg/+^* mice crossed with *APP* Tg2576 mice. Our data indicated that *Arg1* haploinsufficiency promoted Aβ deposition, exacerbated some behavioral impairment, and decreased components of Ragulator-Rag complex involved in mechanistic target of rapamycin complex 1 (mTORC1) signaling and autophagy. Additionally, *Arg1* repression and arginine supplementation both impaired microglial phagocytosis *in vitro*. These data suggest that proper function of *Arg1* and arginine metabolism in myeloid cells remains essential to restrict amyloidosis.

## Introduction

Arginine metabolism associates with numerous biological pathways. The impact of arginine levels and arginine signaling warrants further consideration in Alzheimer’s disease (AD) because of its high demand for multiple metabolic pathways and recent discoveries of mTORC1 activation associated *bona fide* and putative arginine sensors that locate at the lysosome [*SLC38A9* (1), *TM4SF5* (2)], cytoplasm [*CASTORs* (3)], and plasma membrane [*GPRC6A* (4)]. In this respect, properly coordinated arginine metabolism remains critical for linear coupling of signaling events linked to nutrients/amino acids sensing and mTORC1 regulation and could be particularly important for immune cells during neurodegeneration. In addition to arginine’s role as a substrate for protein synthesis and arginine sensors, at least five enzymes catabolize arginine including arginase 1, 2 (*ARG1, ARG2*), nitric oxide synthases 1, 2, 3 (*NOS1, 2, 3*), arginine decarboxylase (*ADC*), arginine/glycine amidinotransferase (*AGAT1*), and arginyltransferase (*ATE1*), suggesting a critical role for numerous biological processes (5, 6). Therefore, *Arg1* may control the degree to which proteolysis occurs, protein turnover, and thus impact proteinopathies. Dysmetabolism of arginine and polyamines were among the most altered in mild cognitive impairment (MCI) and AD patient blood samples (7–9), cerebral spinal fluid (CSF) (9, 10) and postmortem brain tissues (11–14). Alterations in arginine metabolism were also observed in mouse models of Aβ and tau deposition (15–19) and *in vitro* models (20). However, experimental manipulations and animal models concluded different outcomes regarding arginine metabolism in neuropathology (16, 17). Other reports indicated that a potential rare arginase 2 allele was linked to an increased risk of AD (21). These reports signify a relationship between arginine metabolism and AD.

Balanced compartmental arginine levels are necessary for normal physiology and likely require cell type specific demands for proper functioning. Several reports link *Arg1* to microglia/monocyte wound repair and phagocytosis (22, 23). A previous study by our group showed that *Arg1* overexpression decreased neuroinflammation and tau pathology in a tauopathy mouse model (17). Another group reported *Arg1* positive microglia were responsible for reducing Aβ plague deposition during sustained neuroinflammation in an amyloidosis mouse model (24). Monocytes and other peripheral myeloid cells were found to infiltrate the brain and home to Aβ deposits, thus ameliorated amyloidosis pathology (25, 26). However, the impact on Aβ deposition following the myeloid-specific deletion of *Arg1* remains unknown. We determined to assess how the haploinsufficiency of *Arg1* in myeloid cells impacts the brain during amyloidosis using conditional *LysMcre* deletion in *APP* Tg2576 transgenic mice. We performed immunohistochemical analysis, mouse behavioral assessments, and biochemical analysis in mice of amyloidosis and *Arg1* haploinsufficiency. We also performed *in vitro* microglia phagocytosis assays following *Arg1* reduction and arginine supplementation. Our data indicate a critical function for proper arginine and *Arg1* levels during amyloidosis.

## Materials and Methods

### Animal Husbandry

The Tg2576 mice carrying a heterozygous allele for human *APP KM670/671NL* Swedish mutation and non-transgenic (nTg) littermates were bred at the University of South Florida under Dave Morgan and Marcia Gordon. Cre-recombinase mice (B6.129P2-*Lyz2^tm1(cre)/fo^/J; LysMcre* promoter, Stock No: 004781) and *Arg1* mice (C57BL/6*-Arg1^tm1Pmu/^*J (*Arg1^fl^*), Stock No: 008817) were purchased from the Jackson Laboratory. The *APP* Tg2576 mice (APP^+/−^), *Arg1* floxed mice (*Arg1^fl/fl^*), and *LysMcre^Tg/+^* were bred according to the published protocol (27). Therefore, we created four groups including: *nTg/Arg1^+/+^/LysMcre^Tg/+^* (n = 17, nine males/eight females), *nTg/Arg1^fl/+^/LysMcre^Tg/+^* (n = 22, 10 males/12 females), *APP^+/^*^−^*/Arg1^+/+^/LysMcre^Tg/+^* (n = 8, four males/four females, *APP^+/^*^−^*/Arg1^fl/+^/LysMcre^Tg/+^* (n = 10, seven males/three females).

### Behavioral Testing

All mice were aged at 15 months and subsequently exposed to a series of behavioral tasks to evaluate cognition and general activity. All tests were executed in order of increasing stress with appropriate break time between tests to avoid any lingering effects of previous experimental conditions. The experimenter was blinded to the data and experimental groups. The video tracking software ANY-maze (version 4.99, Stoelting Company, Wood Dale, IL) was used.

### Open Field

Open field test (Ugo Basile S.R.L., Italy) was performed to measure general ambulatory activity and anxiety-related behaviors. After a 30-min procedure room acclimation period, mice were allowed to investigate the 44 cm^2^ maze for 15 min while being recorded by ANY-maze. The locomotive activity was measured for total distance traveled and total time immobile. Anxiety-related behavior was measured by time in the center zone. Increased thigmotaxis indicates anxiety-related behavior and suggests an increased anxiety level.

### Y-Maze

The Y-maze test (Ugo Basile S.R.L., Italy) was used to calculate spontaneous alternation to measure spatial working memory and anxiety. Mice were permitted to freely travel the maze (35 cm × 5 cm × 10 cm; L × W × H) for 5 min, while all movements recorded by ANY-maze. Alternation occurs if a mouse entered a different arm during three consecutive entries. The percentage of alternation was calculated (number of alternations divided by the total number of entries minus two). The decreased percentage of alternations suggests impaired working memory.

### Radial Arm Water Maze

We performed a radial arm water maze (RAWM) based on the previous protocol with minor changes (28). In brief, six swimming arms are extending from the center. Only the goal arm contains the escaping platform at the end. Each arm is associated with an obvious visual cue in the surrounding environment. On day 1, mice were trained with constant alternating between visible and hidden platforms with the same goal arm for 15 trials. On day 2, mice were exploring the arms with only the hidden platform located at the same goal arm as day 1 for 15 trials. On day 3 (reversal), the platform was hidden at the goal arm opposite to that used on day 1 and day 2 and mice explored the arms for 15 trials. One error is counted if the mouse explores an arm that is different from the goal arm. An approximate average cohort size of 16 per day was designed and mice in the same cohort ran through each trial sequentially. The average and total errors for three continuous trials are calculated into five blocks over three days.

### Aversive Conditioning

We performed contextual and cued fear conditioning test to measure the animal ability to learn and recollect links between environmental cues and aversive experiences. For this test, an auditory conditioned stimulus (CS, white noise, 70 dB) was accompanied by an unconditioned aversive stimulus (US, a mild foot shock, 0.5 mA) in a novel environment. On day 1 (Training), we placed mice in the fear conditioning apparatus for 180 s, then a 30 s CS was delivered with the US on the metal floor grid during the final 2 s. The training comprised of two CS, paired with two US, with a 2 min interval in between. On day 2 (24 h post-training), we placed mice in the apparatus, with no cues or stimulus (context fear conditioning), for 60 min while monitored for freezing. Directly after contextual testing, we placed the mice into a novel context and exposed them to CS for 180 s (cued fear conditioning). Freezing behavior related to both contextual and cued stimuli were used to assess learning and fear associated recall.

### Immunohistochemistry

We performed immunohistochemistry on free-floating 25 µm sections as previously described (29). At 16 months of age, following all behavioral testing, mice were humanely euthanatized using Somnasol (provided by USF vivarium) and transcardially perfused with 0.9% normal saline. Post perfusion, one hemisphere of the brain was fixed in 4% paraformaldehyde in 100 mM phosphate buffer (pH 7.4) for 24 h while the remaining hemisphere was dissected and stored at −80°C. Cryoprotection was achieved by sequential immersion in 10, 20, 30% of sucrose for 24 h each. Using a sliding microtome, brains were sectioned, placed in DPBS containing 100 mM sodium azide (Sigma-Aldrich, #S2002), and stored at 4°C. The primary antibodies and reagents were used: rabbit anti-*β*-amyloid (Aβ) 1-43 (Covance, #Sig-39145); rabbit anti-Aβ 1-42 (Covance, #Sig-39142); rabbit Aβ 1-40 (Covance, #Sig-39146); rabbit anti-Aβ 1-38 (BioLegend, #808603); rabbit anti-IBA1 (Wako Chemicals, #016-26461); and rat anti-mouse CD68 (AbD Serotec, #MCA1957).

### Detection of Congophillic Deposits

We performed Congo red histology as described previously (30). Briefly, brain sections were mounted and submerged for 20 min in alkaline sodium chloride, then incubated in 0.2% Congo Red solution (Sigma-Aldrich), Congo Red staining kit #HT60-1KT) for 30 min Sections were then rinsed in 95 and 100% ethanol, cleared in xylene for 15 min, and coverslipped with DPX mountant (Electron Microscopy Sciences). Statistical analysis was performed for total, vascular, and parenchymal Aβ loads.

### Protein Preparation for Biochemical Analysis

We prepared protein samples for ELISA and western blotting from the frozen anterior cortex and posterior cortex as previously described (17). Tissues were weighed and resuspended in RIPA buffer (50 mM Tris pH 7.6, 140 mM NaCl, 1% NP40, 0.5% sodium deoxycholate, 0.1% SDS) at 10% wt/vol, containing a 1% vol/vol protease inhibitor cocktail (Sigma-Aldrich, #P8340), phosphatase inhibitor cocktail 2 (Sigma-Aldrich, #P5726), phosphatase inhibitor cocktail 3 (Sigma-Aldrich, #P0044), and PMSF (Sigma-Aldrich, #10837091001). Tissues were mechanically homogenized and sonicated to obtain the whole cell lysate. An aliquot of the whole-cell lysate was centrifuged for 30 min at 40,000 g (4°C). Supernatant detergent soluble (S1) was collected. The resulting pellet became a P1 fraction. The protein concentration of whole-cell lysate and S1 fraction was measured by Pierce™ BCA Protein Assay Kit (Thermo Fisher Scientific, Thermo Scientific™, #23225) according to the manufacturer’s protocol. The P1 protein fraction was resuspended in 70% formic acid using 20% volume of whole-cell homogenate aliquot, then incubated for 1 h at room temperature. Formic acid was buffered with 1M Tris (pH 7.5) using 25% volume of formic acid. A final pH value at 7.5 was adjusted using NaOH solution (50% w/w) to create the detergent insoluble, formic acid (FA) fraction.

### L-Arginine Quantification

Whole-cell lysate samples prepared from the mouse posterior cortex were subjected to liquid chromatography-mass spectrometry/mass spectrometry (LC-MS/MS) using a standard curve of specific amino acid analytes. Quantification of L-arginine was performed by Sanford Burnham Prebys (Orlando, FL, USA).

### Aβ 40 and Aβ 42 ELISA

We measured the concentrations of Aβ 40 and Aβ 42 from the aforementioned detergent soluble S1 and detergent insoluble FA P1 fraction of anterior cortex protein lysate using the Aβ 1-40 and 1-42 specific sandwich ELISA kit (Mega Nano Biotech. FL, USA). In brief, we coated each well of a 96 well plate with 50 µl of goat anti-human Aβ 1-42 antibody (MegaNano BioTech Inc., AB-001) diluted in 1× PBS at 10 μg/ml and incubated overnight at 4°C. We washed the plate five times and blocked it in 200 μl blocking buffer at 37°C for 1 h. After washing the plate, 50 μl diluted detection antibodies anti-Aβ 40 (MegaNano BioTech Inc., Ab40-002) or anti-Aβ 42 (MegaNano BioTech Inc., AB42-002) were mixed with either 50 µl diluted peptide standard solution or diluted samples in a preparation plate before loading to the assay plate. Then we incubated the plates at 4°C overnight. The next day, we washed first and then added 100 µl of diluted secondary antibody into each well and incubated for 45 minutes on an orbital shaker at room temperature. The plate was then washed four times before applying the TMB peroxidase substrate (Surmodics, Inc., TMBS-1000), then the plates were incubated at room temperature for 10 min. The reaction was ended by adding 100 μl/well of 0.4 M H_2_SO_4_. Absorbance at 450 nm was measured with a BioTek Synergy H4 microplate reader. The final concentration was calculated based on peptide standards.

### Western Blotting

We performed western blotting analysis as previously described (17). Briefly, 30 µg of detergent soluble S1 protein was loaded to measure the relative abundance of the protein target. Protein samples were loaded onto the Novex™ 4 to 20% Tris-Glycine Plus 20-well Midi Protein Gels (1.0 mm, Thermo Fisher Scientific, Invitrogen™, #WXP42020BOXA). For a given probing target, all groups of mice were loaded to the same gel including resolving, transferring, and exposure. The following primary antibodies were used: Anti-Liver Arginase (abcam, #ab124917), LAMTOR2 (Cell Signaling Technologies, #8145), LAMTOR3 (Cell Signaling Technologies, #8168), LAMTOR4 (Cell Signaling Technologies, #12284), RagA (Cell Signaling Technologies, #4357), and anti-*β*-actin antibody (Sigma-Aldrich, #A5441). Densitometric analysis was performed using AlphaEase software (Alpha Innoch, CA, US).

### Quantitative Real-Time PCR

We extracted total mRNA from cryopreserved hippocampal tissues using AllPrep DNA/RNA/Protein Mini Kit (QIAGEN, #80004) according to the manufacturer’s protocol. For qRT-PCR analysis, we probed the mouse *Arg1* mRNA from the hippocampal tissues of the aforementioned four mouse groups. The cDNA was generated using the Superscript™ III First-Strand Synthesis System (Thermo Fisher Scientific, Invitrogen™, #18080051). The QuantiTect Primer^®^ Assay was used for mouse *Arg1* gene transcripts (QIAGEN, Mm_Arg1_1_SG, #QT00134288, Lot# 229462537; IDT, Actin, Mm.PT.39a.22214843.g) along with SYBR^®^ Green Quantitative RT-qPCR Kit (Sigma-Aldrich, #QR0100-1KT). We used a standard curve from a pool of all genotypes to span three logs of dynamic range. Melt curve analysis was performed for primer validation. We used the Opticon 2™ Real-Time PCR System (v4.3, Bio-Rad) to detect amplicon.

### Primary Microglial Culture

All cells were maintained in a humidified cell culture incubator (Forma™ Series II 3110, Thermo Fisher Scientific, Thermo Scientific™) with 5% CO_2_ at 37°C. Primary microglia from 6-month-old non-transgenic rats (n = 6, three males/three females) were dissociated as described previously except that we used rat CD11b magnetic microbeads (Miltenyi Biotec, #130-105-634) for the final isolation step (31). Primary microglia were plated at 1.0*10^6^ cells/well on a 6-well plate and maintained in a complete medium using DMEM/F-12 (Thermo Fisher Scientific, Gibco^®^, #11320082), supplemented with 10% heat-inactivated fetal bovine serum (Sigma-Aldrich, #12306C, Lot#17H093), 1% GlutaMAX™-I (200 mM, Thermo Fisher Scientific, Gibco^®^, #35050061), and 1% Penicillin–Streptomycin (10,000 U/ml, Thermo Fisher Scientific, Gibco^®^, #15140122). For arginine supplementation experiments, we added L-arginine (3 mM or 10 mM; Sigma-Aldrich, #W381918-1KG) at 24 h post-plating primary microglia. The control cells were not supplemented with excess L-arginine and kept at the arginine concentration (0.699 mM) in the basal medium. Then 48 h post L-arginine incubation, we performed a pHrodo phagocytosis assay (see below).

### Microglial Cell Line Culture

Highly aggressively proliferating immortalized (HAPI) rat microglial cell line was purchased from MilliporeSigma (#SCC103) and regularly split into a new 75 cm^2^ sterile flask two to three times a week before reaching 80% confluence. HAPI cells were grown in a complete medium using DMEM (Thermo Fisher Scientific, Gibco^®^, #11965167), supplemented with 5% heat-inactivated fetal bovine serum (Sigma-Aldrich, #12306C, Lot#17H093), 1% GlutaMAX™-I (200 mM, Thermo Fisher Scientific, Gibco^®^, #35050061), 1% MEM Non-Essential Amino Acids Solution (100×, Thermo Fisher Scientific, Gibco^®^, #11140050), and 1% Penicillin–Streptomycin (10,000 U/ml, Thermo Fisher Scientific, Gibco^®^, #15140122). For siRNA transfection, we plated HAPI cells at 17 k cells/well on a 24-well plate, then 48 h later when cells were 50–60% confluent, we transfected with either siGLO green oligonucleotide transfection indicator (Dharmacon, #D-001630-01)), non-targeting siRNA (Dharmacon, #D-001210-05-05), or rat specific *Arg1* siRNA (Dharmacon, #M-091161-01-0005) using *Trans*IT-X2^®^ Transfection Reagent (Mirus, #MIR6003) according to the manufacturer’s protocol. We performed the pHrodo phagocytosis assay 48 hours post-transfection.

### Primary Macrophage Culture

Bone marrow derived macrophages (BMDMs) were isolated from the femurs and tibias of *nTg/Arg1^+/+^* mice, *nTg/Arg1^fl/fl^* mice and *nTg/Arg1^fl/fl^/LysMcre^Tg/+^* mice at 3–6 months of age. Bones were flushed with a syringe filled with cold washing media (RPMI 1640 supplemented with 10% FBS and 1% penicillin/streptomycin) to extrude bone marrow into a sterile falcon tube. The bone marrow was then triturated three times using syringes fit with 18 gauge needle and then centrifuged at 1,000 rpm for 5 min at 4°C. After decanting the supernatant, red blood cells were lysed in lysis buffer (0.15 mol/L NH4Cl, 10 mmol/L KHCO3, and 0.1 mmol/L Na2EDTA, pH 7.4) for 3 min. The remaining cells were washed and pelleted in washing media, then resuspended in BMDM differentiation media (RPMI 1640 supplemented with 1% penicillin/streptomycin, 1% HEPES, 0.001% *β*-mercaptoethanol, 10% FBS, and 20% supernatant from sL929 cells-provides macrophage colony stimulating factor) (32). BMDM cells were plated in T75 flasks at 5–8 × 10^5^ cells/ml. Cell culture media was changed on post days of 2, 4, and 6, and then cells were re-plated at the density of 1 × 10^6^ cells/ml on day 7 in differentiation media without sL929 supernatant. The following day, BMDMs were stimulated with LPS (100 ng/ml; Invitrogen) + IFN-gamma (20 ng/ml; eBioscience), IL-4 (20 ng/ml; eBioscience), or LPS (50 ng/ml) + IL-4 (20 ng/ml) in Neuro-2a growth medium or differentiation media without sL929 supernatant as previously described (33). Azithromycin (AZM, Sigma PHR1088, 25 μM) was added to the BMDMs at the time of stimulation. Unstimulated BMDMs were maintained in the appropriate growth medium as controls. 24 h post stimulation, the supernatant of the stimulated macrophages (macrophage conditioned media) was collected and centrifuged to remove the cell debris before being applied to Neuro-2a cells for the measurement of neurotoxicity. BMDM cell lysates were used to assess arginase activity using the QuantiChrom Arginase Assay Kit (Bioassay Systems DARG-200) according to the manufacture’s protocol.

### Neurotoxicity Assay

Neuro-2a cells were cultured in growth media which contains 45% DMEM and 45% OptiMEM Reduced-Serum Medium (Life Technologies) supplemented with 10% FBS and 1% penicillin/streptomycin. Experiments were carried out using Neuro-2a cells within 12 passages. Neurotoxicity was assessed after Neuro-2a cells were seeded in 96-well plates at a density of 2 × 10^5^ cells/ml for 24 h in Neuro-2a growth media. Then Neuro-2a growth media were replaced by different stimulated macrophage conditioned media for 24 h. The cell viability was measured by using MTT assay (Sigma-Aldrich) according to the manufacturer instructions and as described previously (34).

### Phagocytosis Assay

We performed the phagocytosis assay using the pHrodo™ Green E. coli BioParticles™ Conjugate for Phagocytosis kit (Thermo Fisher Scientific, Invitrogen™, #P35366) according to the manufacturer’s protocol. Briefly, we resuspended pHrodo™ E. coli BioParticles^®^ conjugates at 1 mg/ml buffer in sterile PBS (pH 7.4). We replaced the culture medium with the BioParticles^®^ suspension at 50 µl per well. Cells were incubated at 37°C for 90 min. We harvested single live cells using Trypsin-EDTA (0.05%, Thermo Fisher Scientific, Gibco^®^, #25300054) and measured the fluorescence using an Accuri^®^ C6 flow cytometer (BD Biosciences, Serial Number 2986).

### Statistical Analysis

We performed all statistical analyses using SPSS (version 25.0, IBM Corp., Armonk, NY, USA) and generated all graphs using GraphPad Prism (version 8.0.0, GraphPad Software, San Diego, CA, USA). Values were represented as mean ± S.E.M. A two-way ANOVA of 2 × 2 factorial analysis was used to determine simple main effects of *APP* transgene genotype (*APP^+/^*^−^*/LysMcre^Tg/+^ vs nTg/LysMcre^Tg/+^*) and *Arg1* haploinsufficiency genotype (*Arg1^fl/+^/LysMcre^Tg/+^ vs Arg1^+/+^/LysMcre^Tg/+^*) as well as the interaction of the two genotypes, followed by pair-wise comparisons for each genotype. An unpaired Student’s t-test was utilized for two-group comparison (*APP^+/^*^−^*/Arg1^fl/+^/LysMcre^Tg/+^ vs APP^+/^*^−^*/Arg1^+/+^/LysMcre^Tg/+^*), such as for measurements of Aβ, which is not present in non-transgenic animals. Two-way ANOVA followed by Dunnett’s or Sidak’s multiple comparison tests were applied in analyzing experiments using bone marrow derived macrophages.

## Results

### *Arg1* Insufficiency in Myeloid Cells Promotes Diffuse Aβ Deposition

To assess how *Arg1* insufficiency in myeloid cells impacted amyloidosis, we crossbred *LoxP Arg1* (*Arg1^fl/fl^*) mice with transgenic *APP* Tg2576 mice (*APP^+/^*^−^) to generate *APP^(+/^*^−^*^)^*/*Arg1*^(fl/−)^ and *APP*^(−/−)^/*Arg1*^(fl/−)^ (non-transgenic, nTg) mice. These mice were bred with the Cre deleter strain (*LysMcre^Tg/+^*). Mice knocked in allele of *LysMcre* harbor a Cre recombinase gene inserted at the lysozyme 2 gene (*Lyz2*) initial ATG coding sequence. The *Lyz2* promoter targets myeloid lineage cells, mostly macrophages, but also a certain percentage of microglia (35–37). All groups contained one allele of the *LysMcre^Tg/+^*. We established four experimental groups of mice: non-transgenic/*Arg1* sufficient mice (*nTg/Arg1^+/+^/LysMcre^Tg/+^*), non-transgenic/*Arg1* insufficient mice (*nTg/Arg1^fl/+^/LysMcre^Tg/+^*), *APP*/*Arg1* sufficient mice (*APP^+/^*^−^*/Arg1^+/+^/LysMcre^Tg/+^*), and *APP*/*Arg1* insufficient mice (*APP^+/^*^−^*/Arg1^fl/+^/LysMcre^Tg/+^*). We confirmed the deficient arginase 1 activity in primary macrophages of mice with *Arg1^flox^* and *LysMcre^Tg/+^* (**Supplementary Figure S1A, B**).

**Figure 1:**
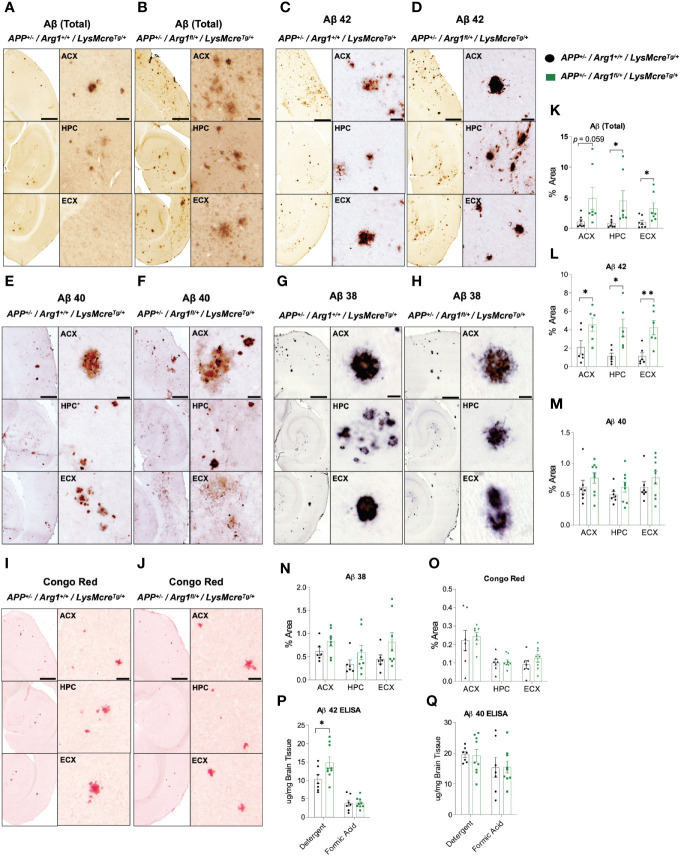
Hemizygous deletion of *Arg1* in myeloid cells of *APP* Tg2576 mice promotes Aβ deposition. We performed immunohistochemistry staining for species of Aβ (total Aβ, Aβ 42, Aβ 40, Aβ 38) and Congophilic Aβ deposition in the anterior cortex (ACX), hippocampus (HPC), and entorhinal cortex (ECX) of 15-month old *APP* Tg2576 mice from two groups (*APP^+/^*^−^*/Arg1^+/+^/LysMcre^Tg/+^*, *APP^+/^*^−^*/Arg1^fl/+^/LysMcre^Tg/+^*). **(A–J)** Representative images for total Aβ **(A, B)**, Aβ 42 **(C, D)**, Aβ 40 **(E, F)**, Aβ 38 **(G, H)**, and Congo red **(I, J)** are presented. **(K–Q)** Quantification analysis of total Aβ **(K)**, Aβ 42 **(L)**, Aβ 40 **(M)**, Aβ 38 **(N)**, and Congo red **(O)** are shown. ELISA was performed for Aβ 42 and Aβ 40 in the cortex. **(P, Q)** Quantification analysis of Aβ 42 **(P)** and Aβ 40 **(Q)** by ELISA for both detergent soluble fraction and formic acid fraction. Quantification of Aβ species and Congo red from staining was determined using six sections per mouse. For mice in each group, n = 6**–**7 for *APP^+/^*^−^*/Arg1^+/+^/LysMcre^Tg/+^*, n = 6**–**9 for *APP^+/^*^−^*/Arg1^fl/+^/LysMcre^Tg/+^*. *p < 0.05; **p < 0.01. Unpaired Student’s t-test. Values represent mean ± SEM. Scale bars represent 500 µm for images and 20 µm for insets.

To determine how *Arg1* insufficiency impacts Aβ deposition, we measured total Aβ, and forms with specific C termini ending at Aβ 38, Aβ 40 and Aβ 42 in the anterior cortex (ACX), hippocampus (HPC), and entorhinal cortex (ECX) of the brain in 16-month-old *APP* Tg2576 mice with *Arg1* sufficiency and insufficiency by immunohistochemistry. Non-transgenic mice had undetectable levels of all Aβ species (data not shown). Overall, total Aβ was increased in HPC (*p* = 0.043), ECX (*p* = 0.034) and trended toward an increase in ACX (*p* = 0.059) in *APP/Arg1* insufficient mice relative to *APP/Arg1* sufficient mice (**Figures 1A, B, K**). Aβ 42 was significantly increased in *APP/Arg1* insufficient mice compared to *APP/Arg1* sufficient mice in all three brain regions (ACX, *p* = 0.038; HPC, *p* = 0.011; ECX, *p* = 0.005) (**Figures 1C, D, L**). However, we did not detect changes in plaque load measured by Aβ 40 (**Figures 1E, F, M**) or Aβ 38 (**Figures 1G, H, N**). Furthermore, we measured compact plaques using the histological stain Congo red and found no changes in the ACX, HPC, and ECX regions between these two groups (**Figures 1I, J, O**). Lastly, we measured Aβ 42 and Aβ 40 by ELISA in both detergent soluble and insoluble (formic acid soluble) fractions. We found increased Aβ 42 in the detergent soluble fraction (*p* = 0.048), but not in the formic acid fraction (**Figure 1P**). No changes were detected for Aβ 40 in ELISA (**Figure 1Q**). Collectively, these data strongly suggest that *Arg1* insufficiency during amyloidosis promotes diffuse Aβ 42 deposition.

### *Arg1* Insufficiency During Amyloidosis Increases Overall Microglial Activation

**Figure 2:**
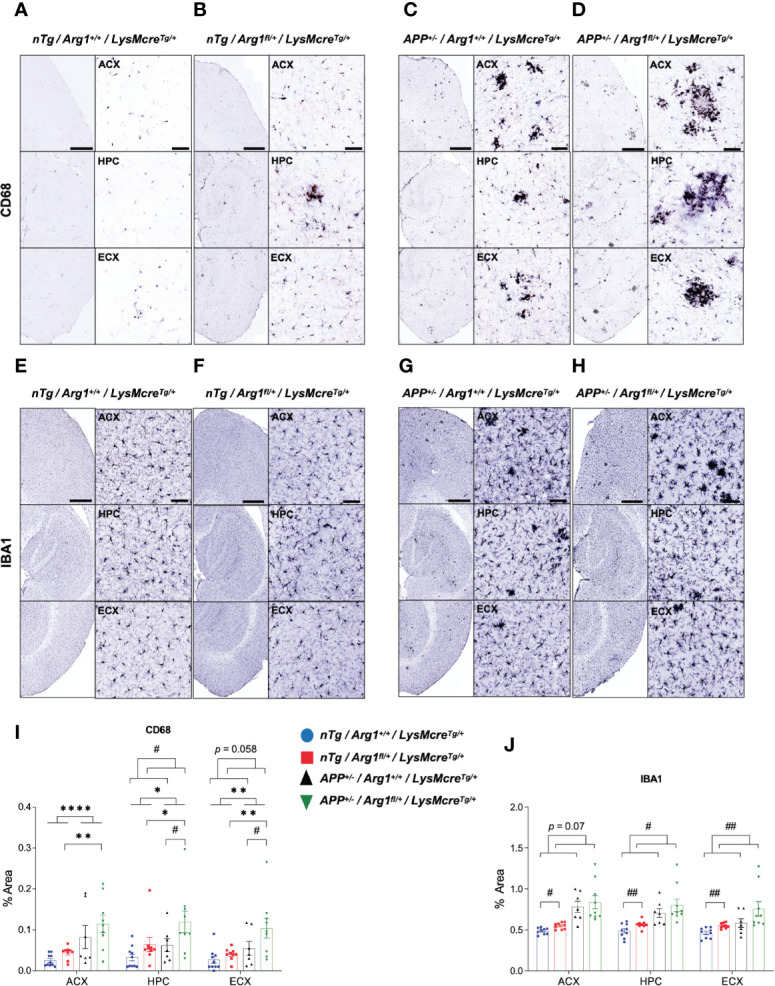
Hemizygous deletion of *Arg1* in myeloid cells increases microglia activation. We performed immunohistochemistry staining for IBA1 and CD68 in the anterior cortex (ACX), hippocampus (HPC), and entorhinal cortex (ECX) of 15-month old mice from four groups (*nTg/Arg1^+/+^/LysMcre^Tg/+^*, *nTg/Arg1^fl/+^/LysMcre^Tg/+^*, *APP^+/^*^−^*/Arg1^+/+^/LysMcre^Tg/+^*, *APP^+/^*^−^*/Arg1^fl/+^/LysMcre^Tg/+^*). **(A–D)** Representative images of IBA1 expression in ACX, HPC and ECX. **(E–H)** Representative images of CD68 expression in ACX, HPC and ECX. **(I)**, Quantification analysis of IBA1 images in **(A–D)**. **(J)**, Quantification analysis of CD68 images in **(E–H)**. Quantification was determined using 6 sections per mouse. For mice in each group, n=9-10 for *nTg/Arg1^+/+^/LysMcre^Tg/+^*, n = 9**–**10 for *nTg/Arg1^fl/+^/LysMcre^Tg/+^*, n = 7 for *APP^+/^*^−^*/Arg1^+/+^/LysMcre^Tg/+^*, n = 9 for *APP^+/^*^−^*/Arg1^fl/+^/LysMcre^Tg/+^*. Asterisk sign (*) denotes statistical significance observed for main effect of *APP* genotype and associated pairwise comparisons. Number sign (#) denotes statistical significance observed for main effect of *Arg1* insufficiency genotype and associated pairwise comparisons. */^#^*p <* 0.05; **/^##^*p <* 0.01; ****p < 0.0001. Two-way ANOVA followed by pairwise comparisons. Values represent mean ± SEM. Scale bars represents 500 µm for images and 50 µm for insets.

Next we measured microglial markers in various regions of the brain by immunohistochemistry during amyloidosis and *Arg1* insufficiency. We stained tissue sections for CD68 (**Figures 2A–D**) and IBA1 expression (**Figures 2E–H**). We found *APP* mice showed a main effect of genotype with higher expression for CD68 in ACX (*p* < 0.0001), HPC (*p* = 0.024) and ECX (*p* = 0.004) compared to nTg mice (**Figure 2I**). Pairwise comparisons between *APP/Arg1* insufficient mice and *nTg/Arg1* insufficient mice were observed (ACX: *p* = 0.003; HPC: *p* = 0.035; ECX: *p* = 0.005; **Figure 2I**). There was a main effect of *Arg1* insufficiency genotype with increased expression for CD68 in HPC (*p* = 0.024) and increased trends for CD68 in ECX (*p* = 0.058) (**Figure 2I**). Particularly, *APP/Arg1* insufficient mice expressed more CD68 in HPC (*p* = 0.042) and ECX (*p* = 0.037) compared to *APP/Arg1* sufficient mice (**Figure 2I**). It also showed either trending or significant main effects of *Arg1* insufficiency genotype for IBA1 in ACX (*p* = 0.07), HPC (*p* = 0.016) and ECX (*p* = 0.008) (**Figure 2J**). The *nTg/Arg1* insufficient mice expressed more IBA1 in ACX (*p* = 0.025), HPC (*p* = 0.006) and ECX (*p* = 0.005) compared to *nTg/Arg1* sufficient mice (**Figure 2J**). While it is well known that Aβ deposition activates microglia, these data also suggest that *Arg1* myeloid insufficiency generally stimulates microglial activation.

### *Arg1* Insufficiency During Amyloidosis Hastens the Impairment of Mouse Behavioral Performance

**Figure 3:**
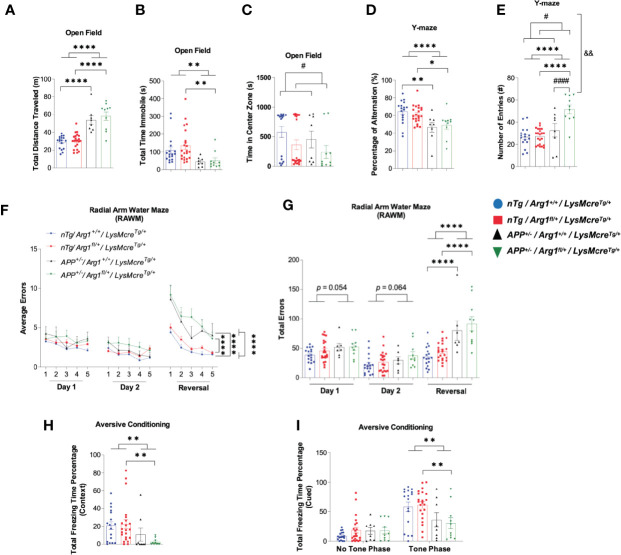
Hemizygous deletion of *Arg1* in myeloid cells of *APP* Tg2576 mice adversely affects behavioral performance. We performed mouse behavioral tests to measure general activity and anxiety in the open field, general working memory in Y-Maze, spatial working memory in Radial Arm Water Maze (RAWM), and fear associated recall in aversive conditioning. The four groups consisted of: *nTg/Arg1^+/+^/LysMcre^Tg/+^*, *nTg/Arg1^fl/+^/LysMcre^Tg/+^*, *APP^+/^*^−^*/Arg1^+/+^/LysMcre^Tg/+^*, *APP^+/^*^−^*/Arg1^fl/+^/LysMcre^Tg/+^*. **(A–C)** Total distance traveled **(A)**, total time immobile **(B)**, and time in the center zone **(C)** were measured and analyzed for open field assessment. **(D, E)** Percentage of alternation **(D)** and the number of entries **(E)** were measured and analyzed for Y-maze assessment. **(F)** Average errors performed by the mice over three days (Day 1, Day 2, and Reversal Day 3) were analyzed for RAWM. **(G)** Total errors performed by the mice on Day 1, Day 2, and Day 3 (Reversal) were analyzed for RAWM. **(H, I)** Percentage of total freezing time from context **(H)** and cued (no tone phase and tone phase) **(I)** were measured and analyzed for aversive conditioning assessment. For mice in each group, n = 17 for *nTg/Arg1^+/+^/LysMcre^Tg/+^*, n = 22 for *nTg/Arg1^fl/+^/LysMcre^Tg/+^*, n = 7**–**8 for *APP^+/^*^−^*/Arg1^+/+^/LysMcre^Tg/+^*, n = 10 for *APP^+/^*^−^*/Arg1^fl/+^/LysMcre^Tg/+^*. Asterisk sign (*) denotes statistical significance observed for the main effect of *APP* genotype and associated pairwise comparisons. The number sign (#) denotes statistical significance observed for the main effect of *Arg1* insufficiency genotype and associated pairwise comparisons. Ampersand sign (&) denotes statistically significant interaction between *APP* genotype and *Arg1* insufficiency genotype. *^/#^*p <* 0.05; **^/&&^*p <* 0.01; ****^/####^*p <* 0.0001. Two-way ANOVA followed by pairwise comparisons. A repeated measure of two-way ANOVA was performed for RAWM average errors **(G)**. Values represent mean ± SEM.

Next, we determined if *Arg1* insufficiency impacts behavioral performance in *APP* and nTg mice. We used open field behavior to measure overall activity by total distance traveled and total immobile time, and anxiety by total time spent in the center zone. The *APP* mice were more active relative to nTg mice measured by increased total distance traveled (*p* < 0.0001, **Figure 3A**) and decreased total time immobile (*p* = 0.001, **Figure 3B**). Although we did not find any difference for the time spent in the center zone in mice with *APP*, we found that mice with *Arg1* insufficiency spent less time in the center, indicating increased anxiety (*p* = 0.039, **Figure 3C**). We measured mouse general working memory by Y-maze and showed that *APP* mice demonstrated a decreased percentage of alternation (*p* < 0.0001), suggesting decreased working memory with amyloidosis (**Figure 3D**). Additionally, we found main effects in genotypes of *APP* (*p* < 0.0001) and *Arg1* insufficiency (*p* = 0.027), as well as an interaction between genotypes in measuring the number of entries in Y-maze (*p* = 0.003). Specifically, the *APP/Arg1* insufficient mice displayed an increased number of entries compared to *APP/Arg1* sufficient mice (*p* < 0.0001), suggesting *APP/Arg1* insufficient mice were more exploratory (**Figure 3E**). Furthermore, we tested mice in the radial arm water maze (RAWM), to measure spatial working memory. Mice received two days of training using one goal arm followed by 1 day of reversal training (using the opposite arm as the goal arm). Errors were counted during training until the mouse reached the rescue platform. Repeated measure ANOVA analysis over three days showed that mice with *APP* performed more errors compared to nTg mice (*p* < 0.0001, **Figure 3F**). Examining each day of training separately revealed that *APP* mice successfully learned procedural aspects of the maze so there were no main genotype effects on Day 1 (*p* = 0.054) or Day 2 (*p* = 0.064) of training (**Figure 3G**). However, *APP* mice were less able to learn a new platform location, so there was a main effect of *APP* genotype on the reversal day of testing (*p* < 0.0001, **Figure 3G**). The total number of errors for *APP/Arg1* insufficient mice was the highest amongst all groups (**Figure 3G**). Lastly, we measured aversive conditioning for fear associated memory. After the initial training trials for all groups, we measured the aversive conditioning by calculating the percentage of freezing time during contextual and cued testing. In the context testing, the *APP/Arg1* insufficient mice showed the lowest percentage of freezing time among all groups, and significantly less than *nTg/Arg1* insufficient mice (*p* = 0.009), which contributed to the overall main effect in *APP* genotype (*p* = 0.006) (**Figure 3H**). In cued testing, all groups of mice showed a higher percentage of freezing time during the toning phase than the no tone phase, indicating an association with learning behavior (**Figure 3I**). During the toning phase, the *APP/Arg1* insufficient mice demonstrated the weakest freezing response among all groups and had less total freezing time percentage than *nTg/Arg1* insufficient mice (*p* = 0.009), which accounts for the main effect in *APP* genotype (*p* = 0.002) (**Figure 3I**). In summary, mice with amyloidosis display impaired behaviors activities, anxiety, and memory, while *Arg1* insufficiency exacerbated these effects.

### *Arg1* Insufficiency Decreases Total Arginase 1 Expression and Increases Arginine in Mouse Brain

**Figure 4:**
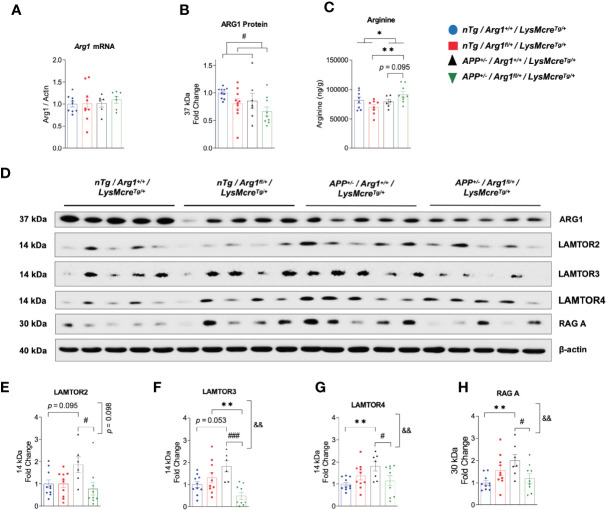
Hemizygous deletion of *Arg1* in myeloid cells reduced total *Arg1* and Ragulator-Rag complex components during brain amyloidosis. We measured *Arg1* mRNA by qRT-PCR, ARG1 protein by western blotting, and its catabolizing substrate arginine by LC-MS/MS. We also measured key Ragulator-Rag complex component proteins critical for microglial phagocytosis by western blotting. Mouse brain samples used were comprised of four experimental groups: *nTg/Arg1^+/+^/LysMcre^Tg/+^*, *nTg/Arg1^fl/+^/LysMcre^Tg/+^*, *APP^+/^*^−^*/Arg1^+/+^/LysMcre^Tg/+^*, *APP^+/^*^−^*/Arg1^fl/+^/LysMcre^Tg/+^*. **(A)** Quantification analysis of qRT-PCR data for *Arg1* mRNA that was normalized to *β*-actin. **(B)** Quantification analysis of western blot data for ARG1 protein. **(C)** Quantification analysis of LC-MS/MS data for amino acid arginine. **(D)** Representative images of western blotting for ARG1, LAMTOR2, LAMTOR3, LAMTOR4, and RAG **(A) (E–H)** Quantification analysis of LAMTOR2 **(E)**, LAMTOR3 **(F)**, LAMTOR4 **(G)** and RAG A **(H)** from images in **(D)**. For mice in each group, n = 8**–**10 for *nTg/Arg1^+/+^/LysMcre^Tg/+^*, n=8-10 for *nTg/Arg1^fl/+^/LysMcre^Tg/+^*, n=6-7 for *APP^+/^*^−^*/Arg1^+/+^/LysMcre^Tg/+^*, n=7-9 for *APP^+/^*^−^*/Arg1^fl/+^/LysMcre^Tg/+^*. Asterisk sign (*) denotes statistical significance observed for the main effect of *APP* genotype and associated pairwise comparisons. The number sign (#) denotes statistical significance observed for the main effect of *Arg1* insufficiency genotype and associated pairwise comparisons. Ampersand sign (&) denotes statistically significant interaction between *APP* genotype and *Arg1* insufficiency genotype. *^/#^*p <* 0.05; **^/&&^*p <* 0.01; ^###^*p <* 0.001. Two-way ANOVA followed by pairwise comparisons. Values represent mean ± SEM.

To determine if hemizygous deletion of *Arg1* in myeloid cells impacts total ARG1 expression and arginine levels in the brain, we measured *Arg1* mRNA by qRT-PCR, ARG1 protein by western blotting and arginine by LC-MS/MS. Interestingly, although we did not find changes in *Arg1* mRNA between groups, we detected an overall decreased expression of ARG1 protein by the main genotype effect of *Arg1* insufficiency (*p* = 0.032) (**Figures 4A, B, D**). We detected a higher level of arginine in the *APP/Arg1* insufficient mice relative to *nTg/Arg1* insufficient mice (*p* = 0.003) and the main effect of *APP* genotype (*p* = 0.036) (**Figure 4C**). We also observed a trend for increased arginine comparing *APP/Arg1* insufficient mice to *APP/Arg1* sufficient mice (*p* = 0.095) (**Figure 4C**). Overall, hemizygous deletion of *Arg1* in myeloid cells modestly decreased total ARG1 expression and increased arginine levels during brain amyloidosis.

### Ragulator-Rag Complex Critical for Microglial Phagocytosis Is Increased During Amyloidosis and Blunted in *Arg1* Insufficient *APP* Mice

Several key studies using different models reported that several components of the Ragulator-Rag complex, namely *LAMTOR2, LAMTOR3, LAMTOR4*, and *RAG A* were essential regulators for maintaining normal lysosomal activity and microglial phagocytosis; loss of functions inhibit phagocytic digestion, even after engulfment of neuronal debris (38, 39). Therefore, we measured the expression of LAMTOR2, LAMTOR3, LAMTOR4, and RAG A by western blot. Densitometry analysis showed that *APP/Arg1* sufficient mice had increased expression in LAMTOR4 (*p* = 0.005), RAG A (*p* = 0.002) and increased trend of LAMTOR2 (*p* = 0.095) and LAMTOR3 (*p* = 0.053) relative to *nTg/Arg1* sufficient mice (**Figures 4D–H**). However, this effect was blocked in *APP/Arg1* insufficient mice compared to *APP/Arg1* sufficient mice. Importantly, the *APP/Arg1* insufficient mice had reduced expression in LAMTOR2 (*p* = 0.031), LAMTOR3 (*p* = 0.001), LAMTOR4 (*p* = 0.022) and RAG A (*p* = 0.012) compared to *APP/Arg1* sufficient mice, thus resulted either trending or significant interactions between *APP* and *Arg1* insufficiency genotypes on LAMTOR2 (*p* = 0.098), LAMTOR3 (*p* = 0.002), LAMTOR4 (*p* = 0.008), and RAG A (*p* = 0.002), respectively (**Figures 4E–H**). Despite increased microglia activation measured by IBA1 and CD68, these data strongly suggest that several components of the Ragulator-Rag complex are up-regulated during amyloidosis and reduced with *Arg1* insufficiency, indicating a potential deficit in microglial digestion machinery.

### *Arg1* Repression and Arginine Supplementation Impair Microglia Phagocytosis *In Vitro*

**Figure 5:**
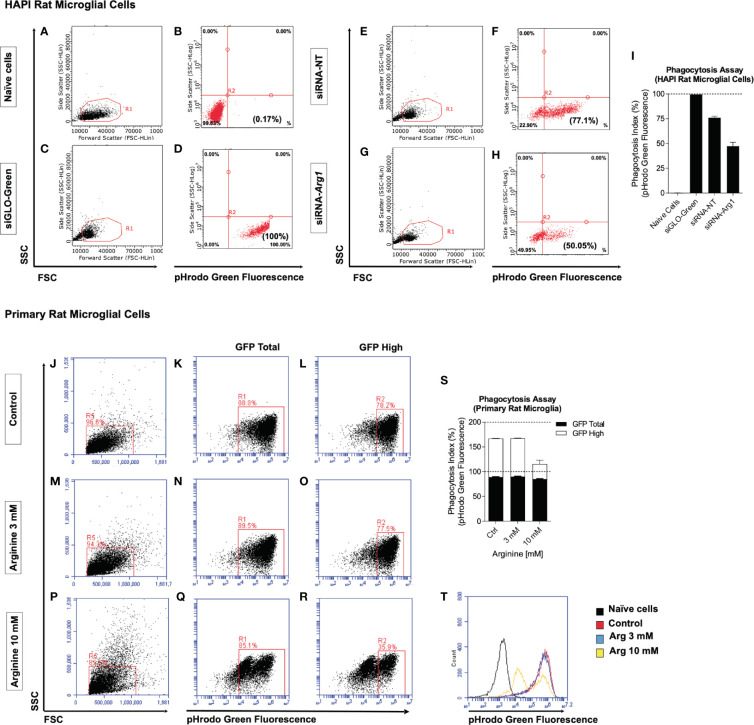
*Arg1* repression by siRNA and arginine supplementation decreases phagocytosis in rat microglial cells. We transfected siRNAs in HAPI rat microglia cells with siGLO-Green, non-targeting siRNA (siRNA-NT), *Arg1* siRNA (siRNA-*Arg1*), and performed pHrodo phagocytosis assay. **(A–H)** Representative flow cytometry gating analysis is shown in dot plots for naïve cells **(A, B)**, siGLO-Green transfected cells **(C, D)**, siRNA-NT transfected cells **(E, F)**, and siRNA-Arg1 transfected cells **(G, H)**. Naïve cells were not treated with fluorescent green *E. coli* bioparticle conjugates and served as a negative population for green fluorescence. Cells transfected with siGLO-Green siRNA were used as positive controls for both siRNA transfection efficiency and green fluorescence. **(I)** Quantification analysis of the percentages of green fluorescence from pHrodo phagocytosis assay. pHrodo phagocytosis assay was also performed using primary rat microglia that were incubated with basal arginine (Control, 0.7 mM), 3 mM arginine, and 10 mM arginine. **(J–R)** Representative flow cytometry gating analysis is shown in the histogram and dot plots for the cells from Control **(J–L)**, 3 mM arginine **(M–O)**, and 10 mM arginine **(P–R)**. **(S)** Quantification analysis of the percentages of pHrodo green fluorescence (GFP Total and GFP High). **(T)** Representative overlapping histogram analysis for each condition. Naïve cells were not treated with fluorescent green *E. coli* bioparticle conjugates and thus served as a negative population for green fluorescence. n = 2 independent experiments. The average values from each group are calculated. Values represent mean ± SD.

Two *in vitro* methods were employed to determine if arginine metabolism impacts microglia phagocytic function measured by pHrodo phagocytosis assay. Fluorescent green *E. coli* bioparticle conjugates are presented to phagocytic cells and green fluorescence increases as pH decrease from neutral cytoplasm to the acidic lysosome. The phagocytic activity of cells is measured based on the acidification of the bioparticles as they are ingested by phagosomes (pH 6.1–6.5) and digested by phagolysosomes (pH 5.0–5.5) (40). We detected green fluorescence by flow cytometry and calculated the phagocytosis index (%) based on the percentage of pHrodo green fluorescence. First, we employed an immortalized rat microglial HAPI cell line due to high endogenous *Arg1* expression. Naïve HAPI cells without siRNAs and bioparticles served as a negative control for green fluorescence detected at an average of 0.2% (**Figures 5A, B, I**). HAPI cells treated with siGLO-Green without bioparticles was designed as a positive control for measuring cellular uptake of siRNAs and green fluorescence, which detected at an average of 100% (**Figures 5C, D, I**). After treating cells with bioparticles, cells transfected with non-targeting siRNA (siRNA-NT) showed an average of 76% phagocytosis index (**Figures 5E, F, I**). However, *Arg1* siRNA (siRNA-*Arg1*) transfected cells only showed a phagocytosis index at an average of 47.3%, much lower than cells transfected with siRNA-NT, suggesting that *Arg1* repression impaired phagocytosis in HAPI rat microglial cells (**Figures 5G, H, I**). Second, we treated primary rat microglia at control (basal arginine concentration at 0.7 mM), 3 mM arginine, and 10 mM arginine in the culture medium followed with pHrodo phagocytosis assay. Cells were gated for total and high pHrodo green fluorescence (GFP Total and GFP High) representing the different extent of phagocytosis activity (**Figures 5J–R**). Although the average of GFP Total phagocytosis index was similar among control (88.9%), 3 mM (89.8%), and 10 mM (85%) of arginine, the average of the GFP High phagocytosis index showed larger differences among control (78.4%), 3 mM (77.8%), and 10 mM (30.5%) of arginine (**Figure 5S**). Thus, we observed a dramatic 47.9% reduction in GFP High phagocytosis index with 10 mM arginine compared to the control condition (**Figure 5S**). Interestingly, 3 mM arginine showed an equivalent GFP High phagocytosis index compared to the control condition (**Figure 5S**). A representative histogram of pHrodo green fluorescence clearly showed only the 10 mM arginine treated cells had two small peaks of GFP fluorescence, indicating fewer bioparticles were delivered to the final stage of phagolysosome (**Figure 5T**). Collectively, reduced arginine metabolism induced either by *Arg1* repression or arginine supplementation impairs microglial phagocytosis *in vitro*.

## Discussion

In the present study, we found that the haploinsufficiency of *Arg1* in myeloid cells in a mouse model of amyloidosis increased Aβ deposition, activated microglia, and impaired behavioral performance. Mechanistic studies suggest that impaired phagocytosis secondary to reduced *Arg1* could contribute to Aβ deposition. Overall, we provided evidence that *APP/Arg1* insufficiency increased more diffuse Aβ deposits measured by total Aβ and Aβ 42, and also stimulated microglial activation *via* CD68 and IBA1. The *Arg1* insufficiency in *APP* mice precipitated more behavior impairment evidenced by more anxiety, more exploratory behavior, and decreased fear associated memory. Importantly, the Ragulator-Rag complex was activated during amyloidosis perhaps as a compensatory response to improve microglial phagocytosis and degradation of Aβ. However, reduced Ragulator-Rag complex in *APP* mice with *Arg1* insufficiency during amyloidosis indicated that arginine metabolism has an unappreciated role in executing this function at the level of the lysosome. Finally, we showed *in vitro* evidence that decreased *Arg1* expression or increased arginine levels could inhibit microglial phagocytosis. Collectively, these data support the overall concept that *Arg1* insufficiency in myeloid cells exacerbates amyloidosis induced neuropathology possibly by activating microglia and impairing phagocytosis.

Two isoforms of arginase consisting of cytosolic *ARG1* and mitochondrial *ARG2* were reported to be increased in the frontal cortex of AD patients (11, 21, 41). Additionally, a rare allele of *ARG2* in males showed increased risk of developing early-onset AD (21). In AD mouse models of amyloidosis, increased *Arg1*, but not *Arg2*, was associated with Aβ deposition (16, 41). Although using DFMO or L-norvaline inhibited arginase 1 and 2 and reduced amyloid deposition in AD mouse models, these compounds both non-specifically inhibit other important targets to reduce amyloid pathology (16, 42). Previously we showed that overexpression of *Arg1* in the CNS of rTg4510 tau transgenic mice decreased neuroinflammation, reduced tau pathology, and myeloid *Arg1* knockout mice signified a distinct microglial phenotype that promoted tau pathology (17). This is consistent with another study that reported *Arg1* positive microglia participated in clearing Aβ plaques during IL-1β mediated inflammatory response (24). In the current study, by using conditional *LysMcre* deletion in *APP* Tg2576 transgenic mice, we are the first to measure the specific effect of reducing *Arg1* in myeloid cells in responding to amyloidosis. Our results showed that reduced *Arg1* levels in LysM positive cells promoted amyloidosis in CNS. Therefore, we argued that the *Arg1* expression in myeloid cells was predicted to be a compensatory response to restrict amyloidosis in AD. The *Arg1* promoted the reparative role of macrophages and studies showed neurodegeneration was accelerated in *Arg1* global deletion and myeloid *Arg1* knockout mice (43). However, although there is a lack of studies in AD mouse models regarding *Arg2*, *Arg2* knockout mice showed neuroprotective effects and reduced neurodegeneration in acute models of retinal injury (44, 45). These studies suggest a distinct role of *Arg2* in CNS diseases (46). We also know that knocking out *Arg1* was lethal in mice due to hyperammonemia caused by arginase deficiency, whereas the *Arg2* knockout mice had intact phenotypes and *Arg1/Arg2* double knockout mice exhibited the same arginase deficiency phenotype as the *Arg1* single knockout mice (47, 48). Although myeloid *Arg2* was initially reported to be less involved in immune responses, recent findings suggest that arginase isoforms in myeloid cells may play crucial regulatory roles in immune response (49, 50). It remains interesting for future studies to investigate the immunological role of myeloid arginase 2 in association with AD.

The lysosome-associated protein CD68 in microglia was often assumed to represent enhanced phagocytosis upon Aβ stimulation in AD (51, 52). However, recent work identified lipid droplet accumulating microglia (LDAM) as having phagocytic deficits but show phagosome maturation among the top regulated pathways from LDAM transcriptome. Importantly, LDAM was found to have upregulated endosomal/lysosomal genes including CD68 in BODIPY positive (lipid droplets) microglia (53). Other reports showed that the Ragulator-Rag complex regulates phagocytic flux in zebrafish, and zebrafish lacking functional Ragulator-Rag complexes (i.e. *LAMTOR4*, *RAG A*) promoted and expanded lysosomal compartments but were unable to appropriately digest neuronal debris (38). Therefore, this urges certain caution in the appearance of increased lysosomal activity/biogenesis or expanded lysosomal components such as CD68 that may actually indicate improper system functioning or uncoupling of the phagocytosis/digestion process. Interestingly, another report identified three members of the Ragulator complex (*LAMTOR2, 3, 4*), *RAG A*, and *NRPL2* (GATOR1 complex) as regulators of phagocytosis (39). The authors showed that CRISPR knockout of Ragulator components (*LAMTOR2, 3, 4*) and *RAG A* inhibited phagocytosis, whereas knockout of *NRPL2* increased phagocytosis (39). In our study, we found increased LAMTOR2, 3, 4, and RAG A in *APP/Arg1* sufficient mice, suggesting that Aβ deposition induces Ragulator complex machinery possibly for enhanced phagocytosis. However, *Arg1* haploinsufficiency in myeloid cells blocked this induction in *APP* mice. Our *in vitro* experimental findings also indicated *Arg1* suppression *via* siRNA in microglial cells reduced phagocytosis, providing potential mechanism for the *in vivo* findings. One result of reduced *Arg1* is increased arginine. We also demonstrated that arginine supplementation in primary microglia reduced phagocytosis. Thus, arginine and *Arg1* levels impact phagocytic/digestive function. Collectively, this argues that amyloidosis triggers expression in key Ragulator–Rag complex components critical for lysosomal digestion and that proper *Arg1* function may be required for this response.

Recent studies with microglia/macrophage markers using single cell RNA-sequencing (scRNA-seq) distinguished brain resident microglia from CNS infiltrated myeloid cells (monocytes/macrophages) (54). The *Lyz2* gene increases in active microglial subpopulations particularly during phases of demyelination/remyelination, and associates with *Trem2* independent microglia activation (55, 56). Since the myeloid marker gene *Lyz2* targets both macrophages and microglia, the observed consequence of exacerbated amyloidosis from the current study were possibly attributed to the deficiency of *Arg1* in both brain resident microglia and CNS infiltrated macrophages. Furthermore, as one of the earliest groups utilizing peripheral myeloid cells (monocytes/macrophages) to restrict amyloidosis in AD mouse models, we and others discovered that peripheral monocytes/macrophages were recruited to the sites of Aβ deposition in the brain to phagocytose Aβ plaques and oligomers (25, 26, 57). Together with resident microglia, infiltrated macrophages promoted degradation of cerebral Aβ (58, 59). Recent studies showed *Arg1* played an essential role in efferocytosis of apoptotic cells in primary microglia/macrophage (60, 61). Consistent with the previous report, our current study aligns with the phagocytic process. Therefore, *Arg1* in brain myeloid cells may have dual roles in removing Aβ plaques by phagocytosis and apoptotic neurons through efferocytosis. Nevertheless, future efforts are warranted to further distinguish the role of *Arg1* in brain resident microglia from infiltrated peripheral macrophages in responding to amyloid deposition.

In the past several years, emerging studies reported the antibacterial enzyme lysozyme 2 was also expressed in neurons in addition to myeloid cells, thus complicating the interpretation of results using *LysM-Cre* mice in studying CNS diseases (62). In one study, researchers found *LysM-Cre*-driven tdTomato expressed in less than 25% of microglia, but also observed *LysM-Cre*-driven *MeCP2* expression in neurons from several brain regions (63). In another study, researchers found *LysM-Cre*-tdTomato positive cells were on average account for less than 30% in both microglia and neurons, but could be exclusively expressed in neurons in certain brain regions (64). Most recently, *LysM-Cre*-tdTomato was confirmed to efficiently target nearly 40% of microglia/macrophages and surprisingly found to also target nearly 10% of neurons (65). Therefore, the expected *LysM-Cre* specificity in microglia/macrophages and the unexpected *LysM-Cre* specificity in neurons indicate two limitations in the current study. Indeed, both neurons and microglia are critically involved in AD pathogenesis.

Firstly, all four groups of mice shared the same *LysMcre^Tg/+^* genetic background in which the endogenous *Lyz2* gene was disrupted due to the insert of Cre recombinase (66). The lysozyme was previously reported to show protective properties in amyloid pathology by interacting with Aβ species to reduce Aβ aggregation, and localizing in sites of Aβ plaques in AD brains (67–69). The lysozyme level was also found increased in the CSF of AD patients, presumably produced mainly by brain myeloid cells (67). This assumption was recently confirmed by studying Aβ plaque-associated microglia using scRNA-seq. Converging studies pointed out that *Lyz2* was one of the commonly induced microglial genes by disease-associated microglia (DAM) and microglial neurodegenerative phenotype (MGnD), suggesting the microglial expression of *Lyz2* played an important role in responding to Aβ stimulus in AD mouse models of amyloidosis (55, 70, 71). These findings argue that *Lyz2* gene is mainly expressed in CNS myeloid cells rather than other CNS cells, which could be confirmed in the summarized RNA-seq data website “The Myeloid Landscape 2” (72). Purified CNS cell types showed both human *Lyz* and mouse ortholog *Lyz2* only significantly expressed in CNS myeloid cells rather than other CNS cell types including neurons (GSE73721, GSE52564, GSE75431). It remains unknown if one allele deletion of *Lyz2* in our study impacts the function of DAM/MGnD microglial signatures in reacting to amyloidosis. However, the same *Lyz2* deficient genotype shared with all mouse groups further lessens this concern.

Secondly, although the exact function of *Lyz2* in neurons is not clear and *Lyz2* is not dominantly expressed in neurons, we cannot exclude the fact that *LysM-Cre* recombination reduced *Arg1* expression in neurons to a certain extent in our study. Previously we reported that overexpressing *Arg1* through pseudotyped AAV9 with neuronal tropism in the hippocampus of rTg4510 tau transgenic mice decreased several neuronal tau pathologies and suggested to increase autophagy through decreased mTORC1 signaling (17). One reason is that overexpression of *Arg1* could deplete cellular arginine and eventually decrease arginine sensing mTORC1 activation. In the current study, we inferred that decreased *Arg1* in *LysM-Cre* neurons could accumulate cellular arginine to activate mTORC1 signaling by arginine sensing mechanisms, thus inhibit autophagy activities. Reduced autophagy in neurons had decreased capacity to degrade intracellular Aβ aggregates, further exacerbated the amyloid pathology in conjunction with *Arg1* deficient myeloid cells. For future studies, myeloid specific mouse models like *Cx3cr1-CreERT2* and *Tmem119-CreERT2* should be applied to target monocytes/macrophages and resident microglia, respectively (73–75).

Although arginine and *Arg1* comprise of a general arginine metabolism pathway, based on the recent identification of amino acid sensors and signaling components, it remains possible that arginine and *Arg1* act through different mechanisms to affect phagocytosis and degradation. For example, differential mechanisms could result from cytoplasmic arginine depletion/repletion, lysosomal acidification, and GPCR extracellular signaling; however, further studies are required to elucidate whether these emerging pathways converge or remain independent. Key findings reported arginine signaling through the lysosomal transporter SLC38A9-Ragulator-Rag-mTORC1 axis and CASTOR1-Ragulator-Rag-mTORC1 axis, both of which activate downstream mTORC1 signaling and inhibit autophagy (76). In our study, *APP/Arg1* insufficient mice showed increased CD68 expression in HPC and ECX compared to *APP/Arg1* sufficient mice and *nTg/Arg1 in*sufficient mice, suggesting that amyloidosis and *Arg1* deficiency promoted CD68 expression. However, given the relationship between ARG1-arginine and arginine-Ragulator-Rag-mTORC1-axis, it is possible that myeloid-specific demands for *Arg1* are required in response to amyloidosis and that uncoupling of phagocytosis/digestion initiates increased Aβ deposition. During certain types of inflammation, myeloid/microglia cells launch *iNOS* and *Arg1* temporally, spatially or even simultaneously, which is thought to provoke either M1 or M2 phenotype to facilitate the clearance of debris and would repair (77). Given this, both enzymes deplete arginine levels, which could essentially promote phagocytosis from both pro and anti-inflammatory states. However, the balance of these enzymes in myeloid/microglial cells in response to amyloidosis may be critical in precipitating the AD phenotype. This is evident in work by Kan et al., which argues that excessive *Arg1* also promotes amyloidosis in a *NOS2* null background (16). Probably, *Arg1* and *NOS2* activities, their respective byproducts, and arginine depletion/repletion are temporally critical regarding microglial response to amyloidosis.

Overall, our findings imply that the haploinsufficiency of *Arg1* in myeloid cells during amyloidosis exacerbates AD-like neuropathology, neuroinflammation, and behavioral deficits. It remains unclear as to the exact mechanism of how *Arg1* or arginine governs phagocytic-digestive function. However, emerging evidence may suggest arginine signaling to mTORC1. Sustained *Arg1* would essentially deplete local arginine levels permitting activation of autophagy through mTORC1 inhibition. Therefore, proper *Arg1* levels in microglia/myeloid cells become critical for activating phagocytic responses during challenges such as amyloidosis. While failure to mount sustained *Arg1* during the Aβ challenge could result in local arginine accumulation and phagocytic dysfunction. Importantly, arginine sensors could serve as new therapeutic targets to rebalance failed phagocytic function during amyloidosis or other disorders associated with immune function.

## Data Availability Statement

The raw data supporting the conclusions of this article will be made available by the authors, without undue reservation.

## Ethics Statement

The animal study was reviewed and approved by University of South Florida and University of Kentucky Institutional Animal Care and Use Committee (IACUC).

## Author Contributions

CM contributed to the design and implementation of the research, *in vitro* cell culture, phagocytosis assay, and wrote the first draft of the manuscript. JH contributed to the design and implementation of the research, performed the statistical analysis, and writing of the manuscript. M-LS and AS contributed to the immunohistochemical analysis for IBA1, CD68, Aβ, and Congo red histology. L-SB contributed to the LC-MS/MS analysis of arginine. L-SB, MW, and RD contributed to the animal behavioral assessments. AK contributed to the mRNA extraction, qRT-PCR analysis of *Arg1* mRNA, and western blot analysis. HL contributed to the western blot analysis, *in vitro* cell culture, and phagocytosis assay. DP contributed to the protein preparation and biochemical analysis. CC and XL contributed to the ELISA analysis for Aβ. MG contributed to the breeding and genotyping of the mice. MO, BZ, JG, and DF contributed to the characterization of different *Arg1^flox^* transgenic mouse lines using primary bone marrow derived macrophages. MG, DM, PB, and DL contributed to the design, conceptualization of the research. DL contributed to the interpretation of the data and writing of the manuscript. All authors contributed to the article and approved the submitted version.

## Funding

Funding for this work is provided by the NIH NIA R21-AG055996 (to DL), NIA R01-AG054559 (to DL), NIA R01-AG051500 (to DM), NINDS R01-NS091582 (to JG), NIAID R01-AI095307 (to DF), Alzheimer’s Association AARGD-16-441534 (to DL) and MNIRGD-12-242665 (to DL), Florida Department of Health Ed and Ethel Moore Alzheimer’s disease (8AZ30) (to DL, PB), and IKBX004214 (to PB). CM is awarded by USF Health Neuroscience Institute Dorothy Benjamin Graduate Fellowship in Alzheimer’s Disease.

## Conflict of Interest

The authors declare that the research was conducted in the absence of any commercial or financial relationships that could be construed as a potential conflict of interest.

## Supplementary Material

Supplementary Figure 1 Primary macrophages with *Arg1* deficiency showed reduced arginase activity and failed to improve neuron viability. **(A)** In macrophage arginase activity assay, primary macrophages isolated from both *nTg/Arg1^+/+^* mice (wild type control) and *nTg/Arg1^fl/fl^* mice (Arg1^flox^ transgenic control) showed increased arginase activity post IL-4 treatment with or without LPS (*p* < 0.0001); however, macrophages of *nTg/Arg1^fl/fl^/LysMcre^Tg/+^* mice did not induce arginase activity in both conditions. In addition, in IL-4 treatment with or without LPS co-stimulation, primary macrophages isolated from *nTg/Arg1^fl/fl^/LysMcre^Tg/+^* mice showed decreased arginase activity compared to macrophages from either *nTg/Arg1^+/+^* mice (*p* < 0.0001) or *nTg/Arg1^fl/fl^* mice (*p* < 0.0001). n = 3 independent replicates from three independent biological replication experiments. **(B)** In measuring Neuro-2a viability using macrophage conditioned medium (MCM), neuroprotection drug azithromycin (AZM) co-incubation with LPS/IFN-γ increased neuron viability compared to LPS/IFN-γ stimulation, a phenomenon only observed using MCM from *nTg/Arg1^+/+^* mice (*p* < 0.0001), but not in *nTg/Arg1^fl/fl^/LysMcre^Tg/+^* mice. In addition, AZM co-incubation with LPS/IL-4 showed increased trend in neuron viability than LPS/IL-4 stimulation using MCM from *nTg/Arg1^+/+^* mice (*p* = 0.051), but not in *nTg/Arg1^fl/fl^/LysMcre^Tg/+^* mice. n = 10 independent replicates from three independent biological replication experiments. ****, *p <* 0.0001; n.s. (not significant), *p* > 0.05. Two-way ANOVA followed by multiple comparisons with Dunnett’s or Sidak’s post-hoc tests. Values represent mean ± SEM.
