# Supplementary figures and images for: Arginase 1 Insufficiency Precipitates Amyloid-β Deposition and Hastens Behavioral Impairment in a Mouse Model of Amyloidosis
Source: Front Immunol. 2021 Jan 14;11:582998. doi: 10.3389/fimmu.2020.582998 (PMC7840571; doi:10.3389/fimmu.2020.582998)

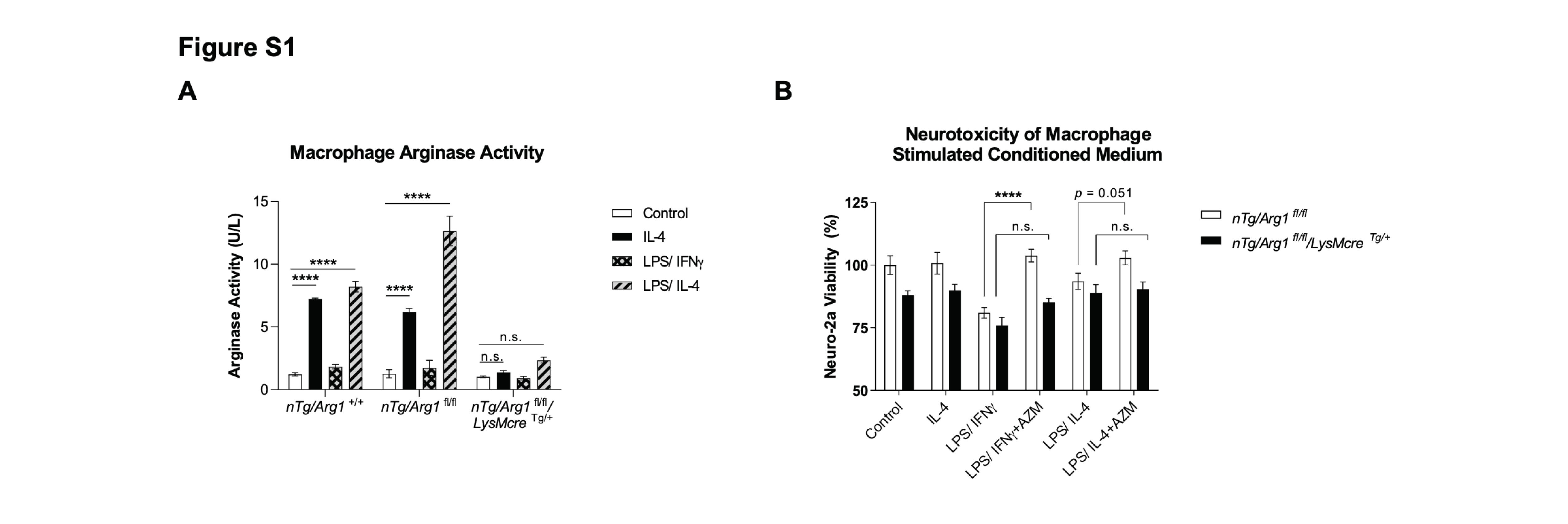

Supplement: Supplementary Figure 1 — Primary macrophages with Arg1 deficiency showed reduced arginase activity and failed to improve neuron viability. (A) In macrophage arginase activity assay, primary macrophages isolated from both nTg/Arg1+/+ mice (wild type control) and nTg/Arg1fl/fl mice (Arg1flox transgenic control) showed increased arginase activity post IL-4 treatment with or without LPS (p < 0.0001); however, macrophages of nTg/Arg1fl/fl/LysMcreTg/+ mice did not induce arginase activity in both conditions. In addition, in IL-4 treatment with or without LPS co-stimulation, primary macrophages isolated from nTg/Arg1fl/fl/LysMcreTg/+ mice showed decreased arginase activity compared to macrophages from either nTg/Arg1+/+ mice (p < 0.0001) or nTg/Arg1fl/fl mice (p < 0.0001). n = 3 independent replicates from three independent biological replication experiments. (B) In measuring Neuro-2a viability using macrophage conditioned medium (MCM), neuroprotection drug azithromycin (AZM) co-incubation with LPS/IFN-γ increased neuron viability compared to LPS/IFN-γ stimulation, a phenomenon only observed using MCM from nTg/Arg1+/+ mice (p < 0.0001), but not in nTg/Arg1fl/fl/LysMcreTg/+ mice. In addition, AZM co-incubation with LPS/IL-4 showed increased trend in neuron viability than LPS/IL-4 stimulation using MCM from nTg/Arg1+/+ mice (p = 0.051), but not in nTg/Arg1fl/fl/LysMcreTg/+ mice. n = 10 independent replicates from three independent biological replication experiments. ****, p < 0.0001; n.s. (not significant), p > 0.05. Two-way ANOVA followed by multiple comparisons with Dunnett’s or Sidak’s post-hoc tests. Values represent mean ± SEM. [file Image_1.tiff]
